# Solubilization of cuprous oxide in water using biosurfactant extracts from corn steep liquor: a comparative study

**DOI:** 10.1038/s41598-022-06386-2

**Published:** 2022-02-17

**Authors:** A. López-Prieto, A. B. Moldes, J. M. Cruz, B. Pérez-Cid

**Affiliations:** 1 Chemical Engineering Department, School of Industrial Engineering – Centro de CINTECX, University of Vigo, Campus As Lagoas-Marcosende, 36310 Vigo, Spain; 2 Food and Analytical Chemistry Department, Faculty of Chemistry, University of Vigo, Campus As Lagoas-Marcosende, 36310 Vigo, Spain

**Keywords:** Biotechnology, Environmental sciences

## Abstract

In this work the chemical characterization (elemental analysis and the content of phospholipids) and surface-active properties of two biosurfactants (BS) extracted with both chloroform or ethyl acetate from corn steep liquor were compared. The phospholipids content for the BS extracted with chloroform (BS1) was considerably higher (8.15%) than that obtained for the BS extracted with ethyl acetate (BS2), 0.11%. When comparing the FTIR spectra of the two BS studied in this work with the spectrum of the commercial surfactant lecithin, a greater similarity (75%) was observed with the spectrum of the BS1. The biosurfactant extract (BS2) provided the most favorable conditions for the solubilization of cuprous oxide (Cu-Ox) in water (12.54% of copper dissolved), in comparison with BS1. The results achieved were considerably better than those obtained with chemical surfactants (Tween 80, SDS and CTAB) on solubilizing Cu-Ox, resulting in the latter cases on percentages of Cu dissolved always lower than 0.21%. In addition, a factorial design was performed obtaining the optimum conditions to solubilize Cu-Ox, where the maximum water solubility of Cu-Ox (13.17%) was achieved using 3.93 g/L of BS2 with a contact time of 19.86 min and using a concentration of Cu-Ox of 1.96 g/L. Thus, the BS2 extract could have a promising future as solubilizing agent in the formulation of more sustainable Cu-Ox based pesticides. Moreover, it was confirmed that the presence of phospholipids prevents the solubilization of copper-based pesticides in water.

## Introduction

Biosurfactants (BS) are biomolecules with a dual hydrophobic/hydrophilic nature that enables to modify the interface between polar and non-polar media, allowing to increase the solubility of polar molecules in non-polar media and vice versa^1^. They are natural molecules produced by a wide variety of microorganisms (bacteria, yeast or fungi) and they can be classified as glycolipids, lipopeptides and high molecular weight BS, including phospholipids and lipoproteins^2^.

Unlike chemical surfactants, BS are more biodegradable compounds with low toxicity, high selectivity, and a critical micellar concentration (CMC) lower than the CMC of chemical surfactants. Hence a lower amount of BS is needed to reduce the surface tension (ST) of water at similar values to those obtained using harmful chemical surfactants. For example, surfactin, a lipopeptide BS produced by *Bacillus subtilis* isolate BS5, can lower the ST of water from about 70 to 36 mN/m, with a CMC around 15.6 mg/L^3^. However, considerably higher results of CMC were found for chemical surfactants, with values around 365 mg/L in the case of hexadecyltrimethylammonium bromide (CTAB)^4^ or 2330 mg/L in the case of sodium dodecyl sulphate (SDS)^5^.

Despite the advantages associated to green surfactants, their production and commercialization is relatively limited due to difficulties associated with low yields in the fermentation processes and high purification costs, resulting in higher prices than chemical surfactants. In order to solve these limitations on the commercialization of BS, the use of inexpensive alternative sources, such as agro-industrial wastes, has been exploited as substrates for the production of BS^1,6^, including vegetable oil processing wastes^7^, fruit and vegetable wastes^8^, lignocellulosic residues^9^, animal wastes^10^, etc.

In the last few years, several studies have been focused on the use of corn steep liquor (CSL), an agro-industrial residue from the corn-milling industry, to obtain a BS that it is produced spontaneously in the residual stream. This multifunctional BS can be extracted with organic solvents from CSL^11^ and it is able to reduce the ST of water between 37.3 and 44.3 mN/m with a CMC between 307 and 400 mg/L^12^, depending on the lot and the organic solvent used during the extraction process. Moreover, the biodegradation capacity of this BS in the environment was also evaluated in a previous work^13^, observing a percentage of biodegradability of 80.7% at 25 °C and pH 7, being much more biodegradable than chemical surfactants.

It is well known that chemical surfactants are widely applied in numerous areas of agriculture including crop protection and agrochemical formulations, where they act as emulsifying, dispersing, spreading and wetting agents enhancing the efficiency of pesticides. However, BS could play an important role in agriculture due to their biological nature and easy degradability^14^. They also have a potential future in the bioremediation of contaminated soils^15^, to remove plant pathogens due to its antimicrobial activity^16^ or even to replace chemical surfactants in pesticide formulations.

The potential of BS in pesticide formulations has a relevant transcendence in sustainable agriculture, preventing the adverse effects caused by harmful pesticides and their chemical associated surfactants, that can remain during several years in surfaces of crops, water and ground water^17^, agricultural soils^18^, etc. Thus, in a previous work, a BS extracted from *Pseudomonas* sp. B0406 was used to enhance the aqueous solubility of two hydrophobic pesticides increasing its availability to fungal mycelia and, consequently, its biodegradation in the environment^19^.

On the other hand, cuprous oxide (Cu-Ox) is a chemical compound widely employed in agriculture to protect plants from pest diseases like blight and downy mildew, although it is poorly soluble in water^20^. In this sense, it could be interesting to use BS from CSL to increase the aqueous solubility of the Cu-Ox pesticide, for improving its biocide efficiency and reduce its persistence in the environment, which supposes an important improvement in the formulation of biopesticides that are more biocompatible with the environment. In this sense, a recent work has evaluated the antimicrobial activity of Cu-Ox nanoparticles stabilized by a lipopeptide biosurfactant^21^.

A recent publication showed that a *Bacillus* strain, *Aneurinibacillus aneurinilyticus*, is responsible for the spontaneous production of BS in CSL^22^. Likewise, Rodríguez-López et al.^23^ have characterized and identified a BS extracted with chloroform from CSL as a lipopeptide composed mainly by C16-C18 fatty acids and amino acids.

However, it is important to highlight that when a BS extract is obtained, this is not constituted by a unique substance but composed by several metabolites, which can be selected using different liquid–liquid (L–L) extraction processes in order to obtain BS extracts with different properties depending on their use. Therefore, in this work, two different L–L extraction processes, based on the use of chloroform or ethyl acetate as organic solvents, were applied to CSL in order to study their selectivity in the extraction of phospholipids and lipopeptides and their effect in the solubilisation of a Cu-Ox pesticide.

## Results and discussion

### Chemical characterization and surface-active properties of the biosurfactants extracted from corn steep liquor

BS used in this work were extracted from CSL using two different organic solvents; chloroform (BS1) and ethyl acetate (BS2); following the methodology described in previous publications^11,24^. In the present work, all BS evaluated have been characterized and identified following the results reported by different analytical methodologies described in the materials and methods section.

Table 1 shows the chemical characterization and surface-active properties of the BS extracted with chloroform (BS1) and ethyl acetate (BS2) from CSL. As shown in Table 1, the BS extracted from CSL were able to reduce the ST of water up to 36.9–38.0 mN/m, which is in concordance with those ST values obtained in previous works for BS extracted from CSL with chloroform^11,23^. Likewise, in this work the ST results obtained for the chemical surfactants were also included and compared with the values obtained for the BS from CSL. SDS reduced the ST of water in more than 30 units, up to 37.7 mN/m, comparably to BS, while all three chemical surfactants gave similar ST values, ranged from 33.7 mN/m for Tween 80 to 34.3 mN/m for CTAB.

**Table 1:** Chemical characterization and surface-active properties of the BS extracted from CSL with chloroform (BS1) and ethyl acetate (BS2).

Analyses	BS1	BS2
Minimum ST (mN/m)	36.9 ± 0.62	38.0 ± 0.31
CMC (mg/L)	424.1 ± 0.44	442.1 ± 0.38
C (%)	74.16 ± 0.01	56.32 ± 0.30
H (%)	11.65 ± 0.16	9.67 ± 0.01
N (%)	1.26 ± 0.01	0.98 ± 0.09
S (%)	< 0.30	< 0.30
Phospholipids (%)	8.15 ± 0.13	0.11 ± 0.00

In addition, as it can be observed in Table 1, the CMC values of all BS extracted from CSL were also evaluated, ranged from 424.1 to 442.1 mg/L for BS1 and BS2, respectively. These results are in concordance with those found in previous works for BS extracted from different CSL, with values ranged from 425.0 to 440.4 mg/L when they were extracted with chloroform or ethyl acetate, respectively^25^. Moreover, CMC of the chemical surfactants was evaluated as well, resulting in values over a wider range than those obtained for BS from CSL. Tween 80 showed a CMC of 15.1 mg/L and CTAB, 360.8 mg/L, and CMC value of SDS was 2371.4 mg/L, resulting, in some cases (SDS), considerably higher than those obtained for all BS employed in this work.

Table 1 shows as well, the chemical characterization (elemental analysis and the content of phospholipids) of the BS extracted from CSL with both chloroform (BS1) and ethyl acetate (BS2). As it can be observed, C and H contents of BS1 were 74.16% and 11.65%, respectively, being higher than those found for BS2, with results of 56.32% of C and 9.67% of H. These results could be attributed to a higher content of phospholipids in the BS extracted with chloroform (BS1), as shown in Table 1, where the content of phospholipids for BS1 was 8.15% and only 0.11% for the BS2. These results were lower than those obtained by Rodríguez-López et al.^23^, where the content of phospholipids resulted in 21% for BS extracted with chloroform from different sources of CSL (FEED and SANTA CRUZ).

**Figure 1:**
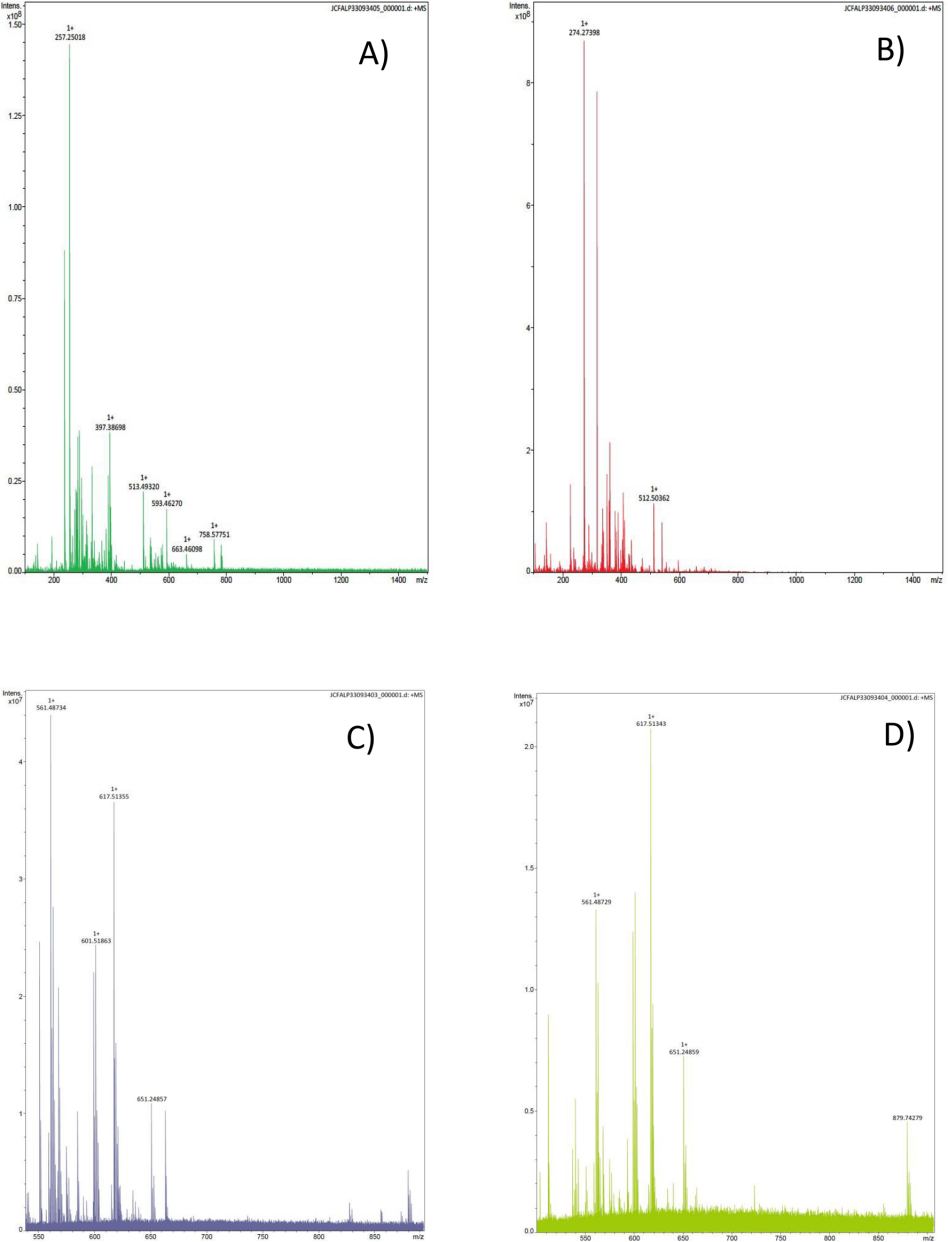
ESI-MS spectra corresponding to the precipitate solution after acetone precipitation of the crude BS extracted from CSL with (**A**) chloroform (BS1) and with (**B**) ethyl acetate (BS2). ESI-MS spectra corresponding to the supernatant solution after acetone precipitation of the BS extract obtained with chloroform (**C**) or ethyl acetate (**D**).

On the other hand, after the phospholipid precipitation, an Electrospray ionization mass spectrometry (ESI-MS) analysis of the precipitate was carried out assessing the composition of the BS studied in this work. Figure 1A and B show both the spectra of the precipitate, of the BS extracted with chloroform (BS1) and with ethyl acetate (BS2) from CSL after acetone precipitation. As it can be observed in Fig. 1A, the spectrum of the precipitate of BS1, revealed a peak at a ratio m/z of 758 Da, which corroborates the presence of phospholipids in the BS extracted with chloroform, resulting in a content of 8.15%, as it was also confirmed by Rodríguez-López et al.^23^ in a recent work. However, this signal was not detected for BS2, which is in concordance with the lower abundance of phospholipids in the BS extracted from CSL with ethyl acetate of 0.11%, as it can be observed in Table 1 and previously mentioned.

**Figure 2:**
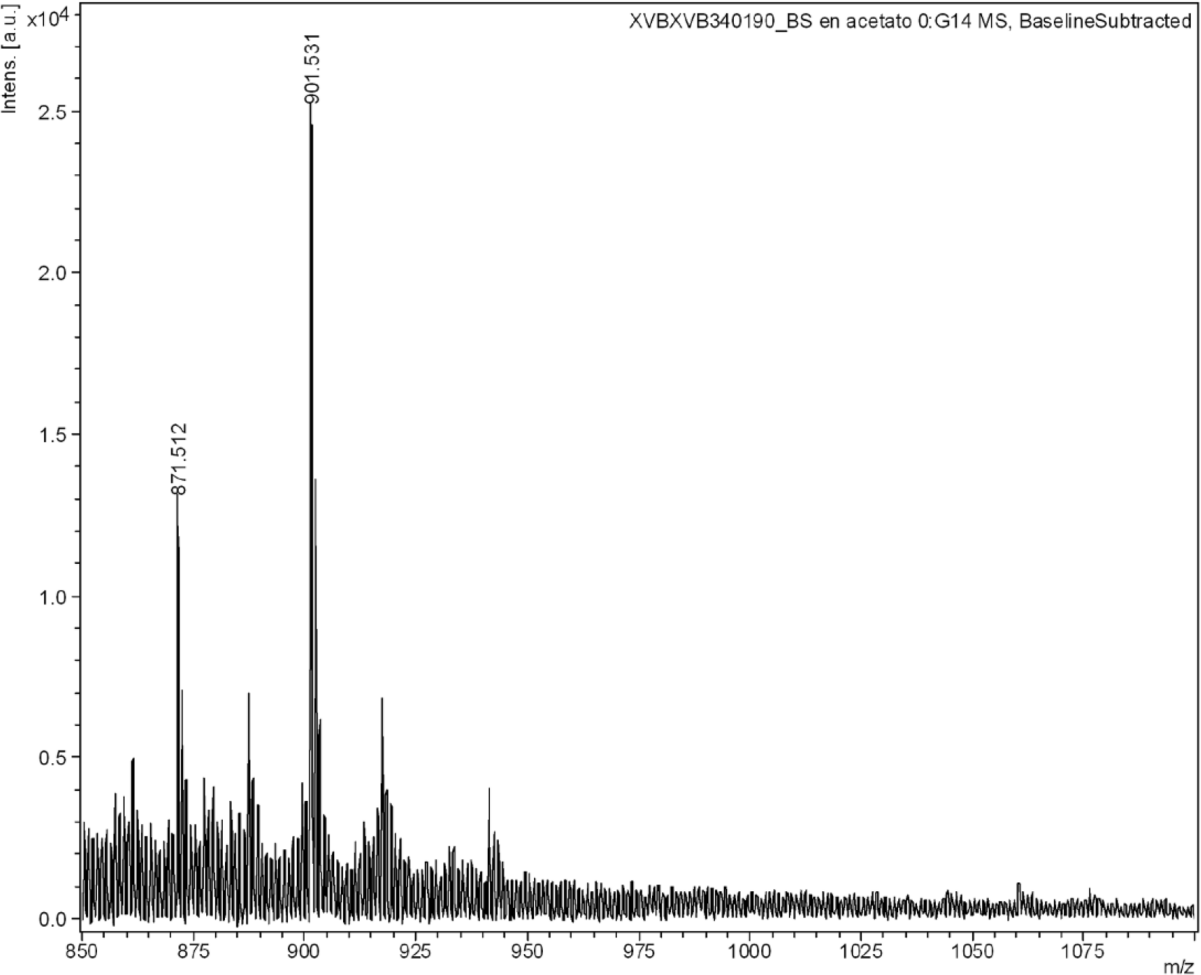
MALDI-TOF MS of the lipopeptide biosurfactant extracted from CSL with ethyl acetate (BS2).

Moreover, Fig. 1C and D show the ESI-MS spectra of the supernatants, after acetone precipitation, for BS1 and BS2 respectively, observing than in Fig. 1C disappeared the mass signal corresponding to phospholipids at 758 m/z. In addition, in the ESI-MS spectra of both supernatants have been observed a molecular mass of 879 m/z that correspond with part of the molecule of the lipopeptide biosurfactant observed by Rodriguez-López et al.^23^ The main lipopeptide contained in the BS extracts possess a molecular weight around 1000 Da similarly to surfactin. Furthermore, in Fig. 2 it is included the mass spectrum of the biosurfactant extract obtained with ethyl acetate from CSL, using matrix-assisted laser desorption/ionization-time of flight mass spectrometry (MALDI-TOF MS) corroborating the presence of the lipopeptide biosurfactant in the extract. In this case the lipopeptide biosurfactant give mass signals of 901 and 871 m/z also observed in the ESI-MS analysis, with a small variation in the mass that it is produced by the differences in the methodology used. The aminoacidic chain and fatty acid chain of this lipopeptide was analysed in a previous work^23^.

**Figure 3:**
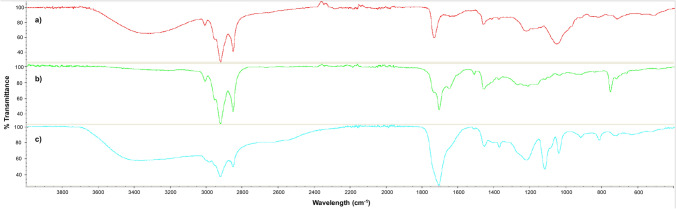
Comparison of FTIR spectra of the commercial surfactant (**a**) lecithin and the BS extracted from CSL with (**b**) chloroform (BS1) and (**c**) ethyl acetate (BS2).

Additionally, a FTIR analysis was conducted to assess the possible similarities between the compositions of lecithin, a commercial BS extracted from egg-yolk, and the BS extracted from CSL with both chloroform (BS1) and ethyl acetate (BS2). As it can be observed in Fig. 3, the FTIR spectra of lecithin (Fig. 3a), gave a similar profile as both BS extracted from CSL, especially BS1 (Fig. 3b), where it was obtained a similarity of 75%, resulting in a strong band between 3000 and 2800 cm^−1^, which indicates the presence of C-CH_3_ bonding, corresponding to long alkyl chains.

On the other hand, lecithin spectrum (Fig. 3a) gave a response of around 41% of similarity regarding BS2 (Fig. 3c). The differences in similarity observed between BS1 and BS2 could be related with a higher presence of phospholipids in the BS1.

### Evaluation of the solubilization capacity of the biosurfactant extracts obtained from corn steep liquor

Data of Cu-Ox solubility at a concentration of 2 g/L of the active principle that was achieved by the BS extracted with chloroform (BS1) or ethyl acetate (BS2) from CSL are also included in Table 2, in comparison with chemical surfactants. Additionally, solubility of both the pure Cu-Ox (97.0% m/m) and commercial fungicide (containing 75% of Cu-Ox) in ultrapure water was also assessed as it is shown in Table 2.

**Table 2:** Effect of BS extracted from CSL with chloroform (BS1) or ethyl acetate (BS2), and chemical surfactants in the percentage of copper dissolved in water from pure cuprous oxide (Cu-Ox; 97.0% m/m) at a concentration of 2 and 20 g/L, in comparison with pure Cu-Ox (Control 1) and Commercial Cu-Ox (Control 2) in absence of surface-active agents.

Surfactant/BS (g/L)	BS1 (%)*	BS2 (%)*	Tween 80 (%)	SDS (%)	CTAB (%)
2	0.50 ± 0.01	12.54 ± 0.13	0.08 ± 0.01	0.05 ± 0.01	0.14 ± 0.01
20	1.63 ± 0.01	6.21 ± 0.50	0.15 ± 0.01	0.21 ± 0.01	0.13 ± 0.01

Control	Control 1 (%)	Control 2 (%)			
	0.09 ± 0.01	0.53 ± 0.04			

*Results of BS(1) and BS(2) were statistically compared, for each concentration level, by *t*-test for data pairs (*p* = 0.05) and significant differences were found between them.

It can be observed that the addition of BS extracted with ethyl acetate (BS2) had a positive effect in the solubilization of Cu-Ox in comparison with the addition of chemical surfactants or the BS extracted with chloroform (BS1). Thus, it is possible to dissolve 12.54% of Cu-Ox when using concentrations of 2 g/L of the BS2, whereas BS1 showed a poor influence on the solubility of Cu-Ox, reaching maximum percentages of solubilization of 1.63% at a concentration of 20 g/L.

The higher capacity of solubilization of a BS extracted from CSL with ethyl acetate was also observed in a previous work with copper oxychloride (Cu-Oxy)^25^, where the results showed values of 96.5% of copper dissolved at a concentration of 16.1 g/L of BS extracted with ethyl acetate from CSL. This increase in the solubility for the BS2 can be attributed to a high abundance of lipopeptides, as it can be observed in Fig. 3c, presenting a more intensive band between 3450 and 3187 cm^−1^ than the BS1 (Fig. 3b). The presence of this pronounced band in the BS extracted with ethyl acetate is related with a higher bioactive fraction, which supposes an enhancement of their surfactant properties^26^. In a previous work, García Reyes et al.^19^ also evaluated the effect of a BS extracted from *Pseudomonas* sp. B0406 on the enhancement of the water solubility of two hydrophobic pesticides (Endosulfan and Methyl parathion) with the aim to significantly increase its availability and promote their biodegradation in the environment**.** This is one of the few works available in the literature that, like to the present work, highlights the use of biosurfactants as a new generation of surfactants and solubilizing agents, different from chemically synthetized ones (some of them constituted by natural polymers produced through chemical reactions combining sugars or fats and inorganic or organic chemical compounds).

Moreover, Table 2 shows, as well, the effect of three different chemical surfactants (Tween 80, SDS and CTAB) on the solubilization of Cu-Ox, resulting in a negligible effect, which is in consonance with the results obtained in a previous work where BS from CSL were used to solubilize Cu-Oxy^25^. The solubility of commercial Cu-Ox pesticide (containing 75% of Cu-Ox) in ultrapure water resulted in 0.53% compared to 0.09% obtained for pure Cu-Ox, which can be attributed to the addition of solubilization agents such as chemical surfactants or other adjuvants in the formulation of commercial fungicides^27^.

As previously commented, the percentage of Cu dissolved in the Cu-Ox/BS2 mixture reached a maximum value of 12.54%, being considerably lower than that found in the Cu-Oxy/BS2 mixture, where it was dissolved around 96.5% of copper^25^. This fact could also be explained by the different interaction established between the BS and both copper-based fungicides (Cu-Oxy and Cu-Ox). In fact, in the case of Cu-Ox, the formation of micelles between the active principle and the BS could be a possibility, whereas in the case of Cu-Oxy it seems more probable that a formation of soluble complexes between the Cu (II) ions and potential ligands found in the BS takes place. In a previous work it was confirmed that the BS extracted from CSL has an amphoteric character^28^ and it is mainly composed by C16–C18 fatty acids and amino acids^23^. These functional groups (amino or acids) have atoms with free electronic pairs with the possibility of forming coordinated or dative covalent bonds, characteristics of metal–ligand complexes^29^.

**Figure 4:**
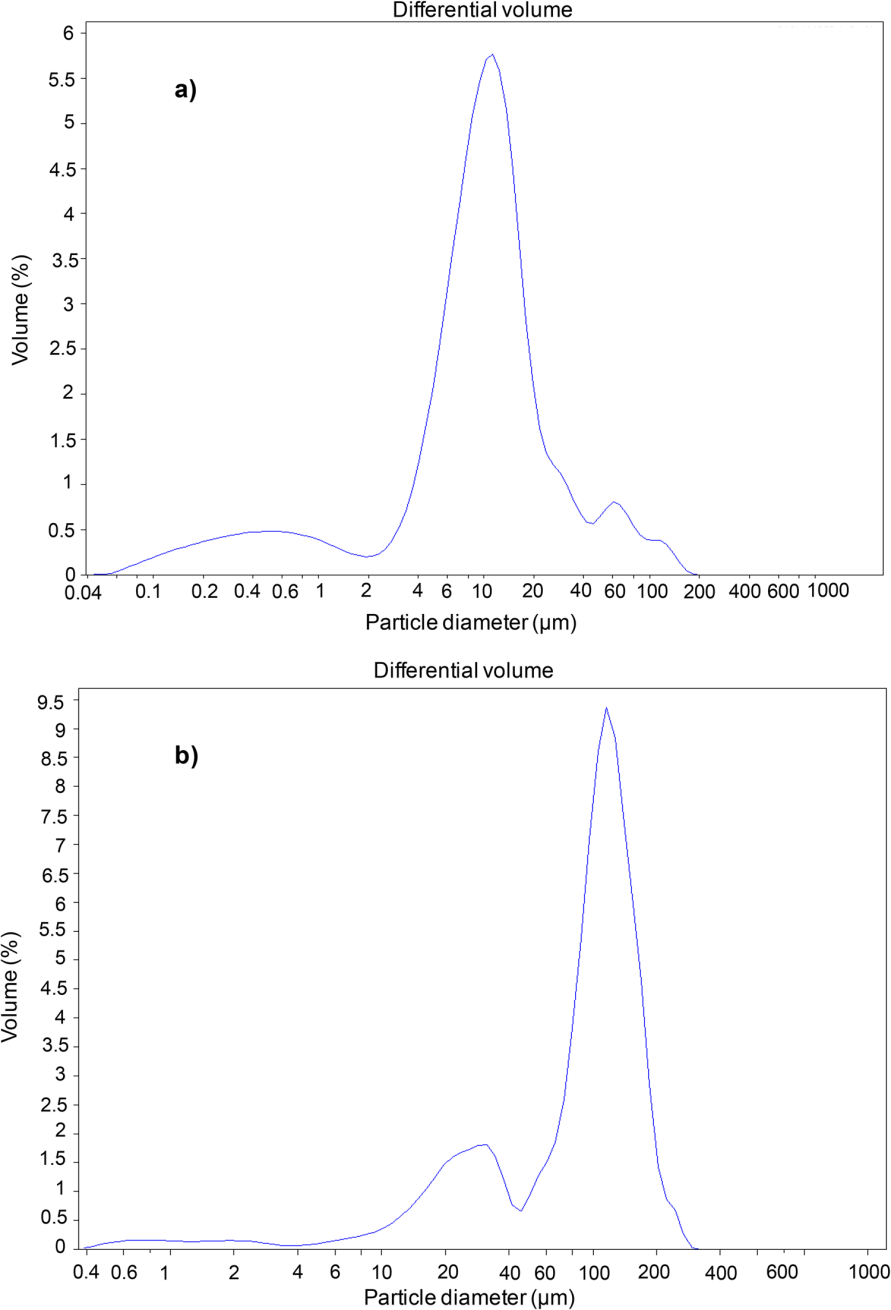
Volume (%) and particle size (µm) distribution in two solutions prepared by mixing copper-based fungicides and a BS extracted from CSL with ethyl acetate (BS2): (**a**) mixture of Cu-Oxy (2 g/L) and BS2 (2 g/L); (**b**) mixture of Cu-Ox (2 g/L) and BS2 (2 g/L).

On the other hand, this theory could be supported by the analysis and distribution of the particle size (µm) in two solutions prepared separately by mixing 2 g/L of each active principle (Cu-Oxy or Cu-Ox) with a fixed concentration of the BS2 (2 g/L) whose results are shown in Fig. 4. In fact, in Fig. 4a corresponding to the mixture Cu-Oxy/BS2, both at a concentration of 2 g/L, it can be observed that between 50 and 90% of the total volume of the particles have a diameter ranged from 9.96 to 30.73 µm, being the mode or the particle diameter with the highest frequency 11.29 µm and the mean value of the particle diameter 15.38 µm. In contrast, in Fig. 4b, corresponding to the mixture Cu-Ox/BS2, both at a concentration of 2 g/L, it was found that between 50 and 90% of the total volume of the particles have a diameter ranged from 102.40 to 163.70 µm, with both mode and mean values of 116.30 and 96.55 µm, respectively. In summary, the diameter of most of the particles found in the Cu-Ox/BS2 mixture is around ten times larger than that found in the Cu-Oxy/BS2 solution, which could be attributed to the formation of micelles (larger particle size) in the first case, contrasting with the formation of metal–ligand complexes in the second case.

According to the results presented until now the BS extracted with ethyl acetate from CSL (BS2) can be considered the best option to solubilize pure Cu-Ox in water in comparison with the BS1 and the other chemical surfactants evaluated.

Therefore, based on these results, an experimental design was developed by optimizing the concentration of BS extracted with ethyl acetate (BS2); coded as *x*_1_; the concentration of Cu-Ox; coded as *x*_2_; and the contact time; coded as *x*_3_ assessing the percentage of copper dissolved from Cu-Ox; coded as *y*_1_. Results showed that the concentration of BS2 (*x*_2_) and the contact time (*x*_3_) were the most statistically significant variables of the factorial design, with *P* values < 0.05. Furthermore, it was observed that the interaction of the concentration of BS2 (*x*_2_) and the contact time (*x*_3_) had a significant effect in the solubilization of Cu.

Table 3 shows the solubility obtained under different conditions following a Box-Behnken factorial design of 15 experiments that after the statistical treatment of data, allowed obtaining a theoretical equation (Eq. 1).

1 $$y_{1} = 7.83 - 1.76x_{2} + 2.34x_{3} - 1.42x_{2} x_{3} - 1.62x_{2}^{2}$$

**Table 3:** Operational conditions used in this study, expressed as coded independent dimensionless variables: concentration of Cu-Ox (*x*_1_), concentration of BS2 (*x*_2_) and contact time (*x*_3_) and the results obtained for the dependent variable, % of copper dissolved (*y*_1_).

Experiment	Independent Variables			Dependent Variable
	*x*_1_	*x*_2_	*x*_3_	*y*_1_
1	0	− 1	− 1	5.372
2	0	1	− 1	4.523
3	0	− 1	1	11.310
4	0	1	1	4.767
5	− 1	− 1	0	7.874
6	− 1	1	0	3.860
7	1	− 1	0	8.151
8	1	1	0	5.504
9	− 1	0	− 1	5.426
10	− 1	0	1	9.741
11	1	0	− 1	4.793
12	1	0	1	13.047
13	0	0	0	7.712
14	0	0	0	7.741
15	0	0	0	8.051

The quadratic equation was composed only by those significant regression coefficients with *P* values < 0.05. The r^2^ value obtained between theoretical and experimental values was 0.947, which suggests that the theoretical equation provided by the model (Eq. 1) can be appropriate to reliably predict the percentage of copper dissolved (*y*_1_), within the ranges established in the experimental design.

**Figure 5:**
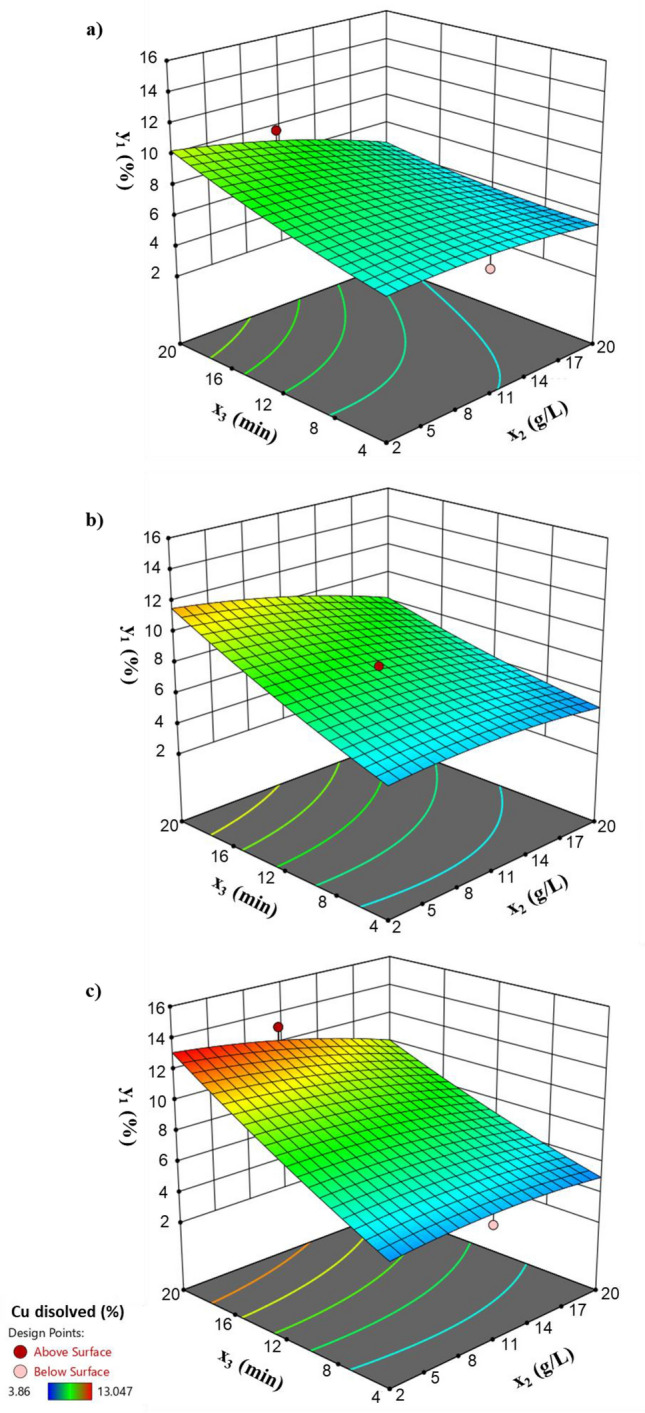
3D response surface-plots showing the variation of the dependent variable (*y*_1_) with the concentration of BS extracted from CSL with ethyl acetate (*x*_2_) and with the contact time (*x*_3_) when using different concentrations of Cu-Ox (*x*_1_): (**a**) 1 g/L, (**b**) 1.5 g/L and (**c**) 2 g/L.

Figure 5 shows the 3D response surface plots associated to the variation of the % of copper dissolved (*y*_1_) with both the concentration of BS2 (*x*_2_) and the contact time (*x*_3_) at different concentrations of Cu-Ox (*x*_1_): (a) 1 g/L, (b) 1.5 g/L and (c) 2 g/L, respectively. As it can be observed, the % of copper dissolved (*y*_1_) increases when increasing the contact time (*x*_3_), being the solubilization of Cu-Ox more effective at low concentrations of BS (*x*_2_), especially at the highest dose of Cu-Ox (*x*_1_), 2 g/L (Fig. 5c). The maximum percentage of copper dissolved (*y*_1_) obtained experimentally was 13.05% at concentrations of Cu-Ox (*x*_1_) of 2 g/L (Fig. 5c). It has not been considered to increase the contact time at values higher than 20 min, when it could not result much operative for agricultural applications, as consumer should prefer those formulations that possess a lower time of preparation. For lower concentrations of Cu-Ox (*x*_1_) of 1 g/L (Fig. 5a) and 1.5 g/L (Fig. 5b), the maximum percentages of copper dissolved (*y*_1_) obtained in the factorial design were around 9.74 and 11.31%, respectively.

In other works, García Reyes et al.^19^ studied a BS extract produced by *Pseudomonas* sp. B0406 that increased the water solubility of Methyl parathion from 34.58 to 48.10 mg/L and the Endosulfan from 0.41 to 0.92 mg/L. Furthermore, another study^30^ also confirmed that the addition of 50 mM calcium chloride to 125 ppm of the Cu-Oxy fungicide was able to significantly increase the water solubility of the active principle from 0.9 to 5.9 µmol/L, after 10 min of contact time.

The maximum percentage of copper dissolved (*y*_1_) predicted by the equation is 13.17% at a concentration of Cu-Ox (*x*_1_) of 1.96 g/L, a concentration of BS2 (*x*_2_) of 3.93 g/L and 19.86 min of contact time (*x*_3_). These results indicate that BS extracted from CSL with ethyl acetate (BS2) could be potentially involved in the formulation of Cu-Ox fungicides, as solubilizing and emulsifying agent and it could also be employed for the removal of copper-based pesticides from soil.

Finally**,** with the aim to assess the predictive capacity of the theoretical equation obtained from the factorial design (Eq. 1), three additional experiments (A, B, C) were conducted. A description of these experiments is revealed in Table 4. They were carried out varying the conditions of the three independent variables within the ranges established but using different values than those employed for the experiments included in Table 3. Experimental results obtained for the trials (A, B, C) are reported in Table 4, and these values were expressed as the mean value and standard deviation of three replicates. In addition, Table 4 shows as well those values estimated by the theoretical model (Eq. 1) for the percentage of copper dissolved (*y*_1_) in the confirmatory assays carried out. All the experiments showed a good agreement between both the experimental data and the values predicted by Eq. (1), being the differences always lower than 1.1%. Moreover, the statistical comparison of the experimental results with the values estimated by the statistical model was conducted by means of a *t* test (*p* = 0.05), and no significant differences were observed in any case. It can be considered, thus, that the theoretical equation (Eq. 1) obtained by the experimental design is highly satisfactory to predict with accuracy reliable results for the dependent variable, (% of copper dissolved, *y*_1_) within the ranges established in the materials and methods—factorial design section for the independent variables (*x*_1_–*x*_3_) studied in this work.

**Table 4:** Validation of the theoretical results estimated by the factorial design for the dependent variable *y*_1_ (% of copper dissolved).

Experiment	Independent variables			Copper dissolved (%)	
	[Cu-Ox] (g/L)	[BS2] (g/L)	Time (min)	Experimental*	Estimated
*A*	2	20	20	6.39 ± 0.42^a^	5.37^a^
*B*	2	2	20	12.83 ± 0.60^b^	11.73^b^
*C*	1	3	19	10.49 ± 0.53^c^	11.27^c^

*Data expressed as mean value and standard deviation of three determinations.

^a,b,c^Values statistically compared by means of *t*-test (*p* = 0.05) and no significant differences were found between them.

## Conclusions

In this work it was confirmed that biosurfactants extracts (BS) obtained from corn steep liquor (CSL), an agro-industrial residue from the corn-milling industry, possess a higher capacity as solubilizing agent of copper pesticides than conventional chemical surfactants, although this capacity vary depending on the nature of copper pesticide, being important to stablish individual studies for each active principle. The positive effect of the BS in the solubilization of Cu-Ox was clear, although lower than the results observed with Cu-Oxy. Comparing both pesticides, the results obtained were in consonance with the lower size distribution particle observed in those formulations containing Cu-Oxy in comparison with formulations containing Cu-Ox in presence of BS. The formation of metal–ligand complexes between Cu-Oxy and BS could promote the solubilization of this active principle. Moreover, BS extracts with higher content in phospholipids produced lower solubilities of Cu-Ox solutions. These results obtained are important and novelty as they demonstrate the importance of individual studies for each active principle. In addition, these results could promote the potential application of the BS extract, obtained with ethyl acetate from CSL, as solubilizing agent in the formulation of more ecofriendly copper-based pesticides for sustainable agriculture.

## Materials and methods

### Extraction of biosurfactants from corn steep liquor: surface-active properties

The extraction of BS from CSL was performed following the methodology described by Vecino et al.^11^ CSL was provided by Nutropam CSL (Companhia Portuguesa de Amidos, S.A. (COPAM), San João da Talha, Portugal). BS were extracted using two different organic solvents: chloroform (CSL solution 1:2 v/v at 56 °C for 60 min) and ethyl acetate (CSL solution 1:3 v/v at room temperature for 60 min). Both solvents were supplied by CARLO ERBA Reagents, S.A.S (Val de Reuil Cedex, France). After extraction, chloroform and ethyl acetate were recovered by vacuum distillation. Moreover, the surface-active properties of the BS extracts were measured based on the ST reduction produced in water in presence of the extracts using a Krüss K20 EasyDyne tensiometer with a 1.9 cm platinum Willhemly plate (Krüss GmbH, Hamburg, Germany) at room temperature. In addition, the minimum concentration of BS that produces the maximum reduction in the ST of water, CMC, was also determined, for that several dilutions of the BS extracts from CSL were prepared starting at concentrations of 1 g/L. All determinations were conducted by triplicate at room temperature.

### Precipitation and quantification of phospholipids

The presence of phospholipids in crude BS extracts obtained from CSL (BS1 and  BS2) was evaluated. Phospholipids were precipitated with acetone following the methodology reported by Choudhary et al.^31^, where about 1.50 g of each of the BS extracts from CSL were mixed with acetone (99.8%, provided by CARLO ERBA Reagents S.A.S, Val de Reuil Cedex, France) in a ratio 1:30 (w/v) and maintained at − 80 °C overnight. Then, the precipitate formed was separated by vacuum filtration, washed with cold acetone and brought to dry in a stove (Incubator IN110, Memmert GmbH + Co. KG, Schwabach, Germany) at 105 °C for 24 h. Finally, the dry weight of the solid residue was quantified, by triplicate, for each analyzed sample.

### Electrospray ionization mass spectrometry/collision-induced dissociation (ESI-MS/CID) analysis

After the phospholipid precipitation, an ESI analysis of both the precipitate and the supernatant fractions, after acetone precipitation of the crude BS extracted from CSL with both chloroform (BS1) and ethyl acetate (BS2), was carried out. For that, 1 mg of the sample was diluted in chloroform or ethyl acetate, as appropriate, and volatilized under vacuum. Then, a current of electrons was used to fragment and ionize the molecules, and the fragmentation patterns were recorded on a mass spectrometer in positive mode (Bruker FTMS APEXIII, Fremont CA).

### Analysis of the biosurfactant extract by MALDI-TOF MS

BS2, extracted with ethyl acetate, was analyzed by matrix-assisted laser desorption/ionization-time of flight mass spectrometry (MALDI-TOF MS), following the experimental procedure described in a previous work slightly modified in the sample preparation^32^. The dried-droplet sample preparation technique was used, applying 2 µL of α-cyano-4-hydroxycinnamic acid (CHCA) matrix solution (10 mg/mL in 50% ethanol/50% acetone, v/v) directly on a MTP AnchorChip 800/384 TF MALDI target (Bruker Daltonik, Bremen Germany). Then, before drying the matrix solution, 2 µL of sample (diluted 1/10 in ethanol) was added and allowed to dry at room temperature. External mass calibration was performed with a calibration standard (Bruker Daltonik, Bremen Germany) for the range m/z 700–4000 (9 mass calibrant points): 0.5 mL of calibrant solution and CHCA matrix previously mixed in an Eppendorf tube (1:2, v/v) were applied directly on the target and allowed to dry at room temperature. Mass spectra were recorded using an Autoflex III smartbeam MALDI-TOF mass spectrometer (Bruker Daltonik, Bremen, Germany), operating in reflector positive ion mode. Ions formed upon irradiation by a smartbeam nitrogen laser (337 nm) using an accelerating potential of 20 kV and a frequency of 200 Hz. Averaging 2000 laser shots collected across the whole sample spot surface by rastering in the range m/z 400–4000 produced each mass spectrum. The laser irradiance was set to 40–60% (relative scale 0–100) arbitrary units. Low molecular ion gating was set to 390 Da to remove the ions below this value arising from the matrix and their clusters or other unknown contaminants. All spectra were acquired and treated using the FlexControl 3.0 and FlexAnalysis 3.0 softwares (Bruker Daltonik, Bremen, Germany), respectively.

### Chemical characterization of biosurfactants from corn steep liquor and lecithin by fourier-transform infrared spectroscopy (FTIR)

BS1 and BS2 samples were subjected to FTIR analysis. In addition, for comparative purposes, lyophilized lecithin (99% m/m), supplied by Sigma Aldrich and obtained from egg yolk was included in the study. Samples of commercial lecithin and BS were ground and pressed with 10 mg potassium bromide (7500 kg for 30 s) to obtain translucent pellets. Infrared absorption spectra of lecithin and the two BS obtained from CSL, BS1 and BS2, were recorded on a FTIR system (Nicolet 6700, Thermo Scientific) with a spectral resolution of 4 cm^−1^ and wavenumber accuracy between 400 and 4000 cm^−1^. As a background reference, a potassium bromide pellet was used in the evaluation of all measurements, obtaining 32 scans per spectrum.

### Solubilization of cuprous oxide in presence and absence of biosurfactants extracted from corn steep liquor

Several surfactant solutions, at concentrations of 2 or 20 g/L of BS extracted from CSL with chloroform (BS1) or ethyl acetate (BS2), were prepared. In this study, for comparative purposes, three chemically synthetized surfactants were also included: Tween 80, SDS and CTAB, and subjected to the same assay.

Preliminary solubility experiments were carried out in 50 mL capacity plastic tubes, with a fixed concentration of Cu-Ox (2 g/L), and a final sample volume of 15 mL. The concentration of Cu-Ox used was the highest dose recommended to be applied in most agrochemical applications^33,34^. Additionally, the pure Cu-Ox (97.0% m/m) and the commercial fungicide (Nordox 75 WG, manufactured by Nordox Indusstrier, A.S. Oslo Norway), containing 75% Cu, were also used at a concentration of 2 g/L in ultrapure water to evaluate the releasing of copper ions. Samples containing the surface-active agents were stirred for 10–15 s in a Vortex, and introduced in a shaker at room temperature during 20 min. Following, the tubes were centrifuged for 10 min at 5000 rpm and 5 °C. The supernatant solutions were filtered with polytetrafluoroethylene (PTFE) membrane filters (0.45 µm) and stored at 4 °C until analysis. All the experiments were carried out by triplicate, and the results are reported as mean value and standard deviation of three determinations.

### Quantification of copper ions

Once the experiments were performed, the content of dissolved copper in the aqueous solutions after the centrifugation step was quantified, to determine the most favourable water-soluble conditions of the Cu-Ox pesticide. For copper determinations, a double-beam flame atomic absorption spectrophotometer (Thermo Scientific, iCE 3000 series) equipped with an air-acetylene flame and with a hollow cathode lamp as radiation source was used. Instrumental parameters like resonance line (324.8 nm), slit width (0.5 nm) and lamp intensity were optimized according to the manufacturer recommendations. Working standards solutions were prepared daily by adequate dilution of the stock copper solution (1000 ± 2 mg/L) supplied by Fisher Chemical. All the measurements were made by triplicate, and the average values were considered. The percentage of dissolved copper was calculated following the Eq. (2):

2 $$Dissolved\;{\text{Cu}} \left( \% \right) = \frac{C \cdot V}{{W \cdot 0.888}} \cdot 100$$

where C is the copper concentration quantified in the aqueous solution (mg/L), V is the volume of the aqueous solution (L); W is the mass of Cu-Ox weighted (mg); the factor 0.888 associated to the ratio between Cu/Cu-Ox.

### Particle size measurement in aqueous solutions of copper-based pesticides

Volume (%) and particle size (µm) distribution were evaluated in aqueous solutions prepared by mixing 2 g/L of copper oxychloride (Cu-Oxy) (99.0% m/m; provided by LGC Labor GmbH, Augsburg, Germany) or Cu-Ox with 2 g/L of the BS extracted with ethyl acetate from CSL (BS2). Analysis was assessed by light scattering in the particle size range from 40 nm up to 2000 µm using a particle size analyzer model LS 13 320 (Beckman Coulter, Spain).

### Factorial design studying the solubilization of cuprous oxide in presence of a lipopeptide biosurfactant extracted with ethyl acetate from corn steep liquor

A deeper study of the effect of BS extract from CSL obtained with ethyl acetate (BS2) on the solubilization of copper ions was carried out by means of an incomplete Box–Behnken factorial design^35^, which allows obtaining a predictive equation. The experiments were carried out following the procedure explained in the solubilization of cuprous oxide in presence and absence of biosurfactants extracted from corn steep liquor and in the quantification of copper ions sections.

The independent variables selected in this study were: Cu-Ox concentration (Cu-Ox); coded as *x*_1_; concentration of BS2; coded as *x*_2_; and contact time (t); coded as *x*_3_, while the dependent variable was the percentage (%) of copper dissolved*,* coded as *y*_1_. The limits established for the concentrations of Cu-Ox (*x*_1_) were ranged from 1 to 2 g/L; the concentrations of BS2 (*x*_2_) were ranged from 2 to 20 g/L and for the contact time (*x*_3_), limits were ranged from 4 to 20 min. The range of the independent variables selected were chosen based in a previous work^25,33,34^.

The independent variables were assigned values representing the operational conditions of minimum limit (-1), central point (0) and maximum limit (1) according to the range of values established previously for each variable as it is shown in Table 3.

The experimental data were analyzed by means of the response surface method with the Design-Expert Version 12 software (Stat-Ease, Inc., Minneapolis, MN, USA), obtaining a quadratic equation (Eq. 1) relating the dependent variables and regression coefficients in order to obtain the results of the dependent value for each condition within the range established previously as it has been performed in a previous publication^25^. This equation allows predicting the percentage of copper dissolved within the range of the independent variables considered in the factorial design.

Additionally, the theoretical equation provided by the model was validated by means of three additional specific trials (named A, B and C). These additional experiments were carried out following the same procedure described in the solubilization of cuprous oxide in presence and absence of biosurfactants extracted from corn steep liquor and in the quantification of copper ions sections, but varying the independent variables within the intervals established in the experimental design and always using different operational conditions than those indicated in the 15 experiments of the Box-Behnken factorial design described in Table 3. Each verification experiment was conducted by triplicate and the results were reported as the mean value and standard deviation of three determinations. In order to evaluate the predictive capacity of the theoretical equation, the experimental results were statistically compared with those estimated by the model.
